# Carbohydrate-Binding Properties and Antimicrobial and Anticancer Potential of a New Lectin from the Phloem Sap of *Cucurbita pepo*

**DOI:** 10.3390/molecules29112531

**Published:** 2024-05-27

**Authors:** Md. Aminul Islam, Md. Mikail Hossain, Alima Khanam, A. K. M. Asaduzzaman, Syed Rashel Kabir, Yasuhiro Ozeki, Yuki Fujii, Imtiaj Hasan

**Affiliations:** 1 Department of Biochemistry and Molecular Biology, University of Rajshahi, Rajshahi 6205, Bangladesh; aminulislam139500@gmail.com (M.A.I.); mikailhossain97@gmail.com (M.M.H.); alima.bmbru@gmail.com (A.K.); jonyasad2005@ru.ac.bd (A.K.M.A.); rashelkabir@ru.ac.bd (S.R.K.); 2 Graduate School of Nanobiosciences, Yokohama City University, 22-2 Seto, Kanazawa-ku, Yokohama 236-0027, Kanagawa, Japan; ozeki@yokohama-cu.ac.jp; 3 Graduate School of Pharmaceutical Sciences, Nagasaki International University, 2825-7 Huis Ten Bosch, Sasebo 859-3298, Nagasaki, Japan; yfujii@niu.ac.jp; 4 Department of Microbiology, University of Rajshahi, Rajshahi 6205, Bangladesh

**Keywords:** *Cucurbita* lectins, chitin binding, bacteriostatic, antibiofilm, antifungal, antitumor

## Abstract

A Cucurbita phloem exudate lectin (CPL) from summer squash (*Cucurbita pepo*) fruits was isolated and its sugar-binding properties and biological activities were studied. The lectin was purified by affinity chromatography and the hemagglutination assay method was used to determine its pH, heat stability, metal-dependency and sugar specificity. Antimicrobial and anticancer activities were also studied by disc diffusion assays and in vivo and in vitro methods. The molecular weight of CPL was 30 ± 1 KDa and it was stable at different pH (5.0 to 9.0) and temperatures (30 to 60 °C). CPL recovered its hemagglutination activity in the presence of Ca^2+^. 4-nitrophenyl-α-D-glucopyranoside, lactose, rhamnose and *N*-acetyl-D-glucosamine strongly inhibited the activity. With an LC_50_ value of 265 µg/mL, CPL was moderately toxic and exhibited bacteriostatic, bactericidal and antibiofilm activities against different pathogenic bacteria. It also exhibited marked antifungal activity against *Aspergillus niger* and agglutinated *A. flavus* spores. In vivo antiproliferative activity against Ehrlich ascites carcinoma (EAC) cells in Swiss albino mice was observed when CPL exerted 36.44% and 66.66% growth inhibition at doses of 3.0 mg/kg/day and 6.0 mg/kg/day, respectively. A 12-day treatment by CPL could reverse their RBC and WBC counts as well as restore the hemoglobin percentage to normal levels. The MTT assay of CPL performed against human breast (MCF-7) and lung (A-549) cancer cell lines showed 29.53% and 18.30% of inhibitory activity at concentrations of 128 and 256 µg/mL, respectively.

## 1. Introduction

Lectins are a widely distributed class of proteins or glycoproteins that agglutinate erythrocytes and bind carbohydrates (glycans) [1]. They are neither immunoglobulins nor enzymes, and their ability to recognize and bind specifically to carbohydrate ligands without any chemical modification sets them apart from other carbohydrate-binding proteins and enzymes, making them an invaluable tool in biomedical and glycoconjugate research [2,3]. On a cellular and molecular level, lectins play critical roles in the recognition of cells, proteins and carbohydrates [4,5]. Depending on their binding specificity, lectins have been used as one of the most powerful reagents for sugar chain detection in biochemical and histochemical inquiry [6]. In plants, lectins are a distinct class of proteins that exhibit significant biological properties such as agglutination, toxicity, the inhibition of the growth of cancer cells and antimicrobial properties [7,8,9].

In the plant communication system, the phloem is a dynamic transport system. Phloem sap is composed of sugars, vitamins, and organic and inorganic acids as well as many structurally different proteins, referred to as phloem proteins (P-proteins) [10]. Biological functions of P-proteins include wound sealing and defense responses against insects and herbivores through the phloem-based defense mechanism. During any stress-like insect attack, several proteins including lectins get expressed and interact with pathogen-associated molecular pattern (PAMP) and damage-associated molecular pattern (DAMP) molecules to perform protective functions in plants [11]. 

Squash is one of the oldest known crops [12]. *Cucurbita pepo*, commonly known as zucchini or summer squash, is a versatile and economically significant plant species belonging to the gourd family, Cucurbitaceae. In this family, the major protein fraction of phloem exudate consists of phloem proteins 1 and 2 (PP1 and PP2). Many of these PP2s are lectins, known as Cucurbitaceae phloem exudate lectins (CPELs), which have been purified from the phloem of many Cucurbitaceae fruits like cucumber (*Cucumis sativus*), pumpkin (*Cucurbita maxima*), squash (*Cucurbita pepo*), melon (*Cucumis melo*), ridge gourd (*Luffa acutangula*), ivy gourd (*Coccinia indica*), snake gourd (*Trichosanthes anguina*), bottle gourd (*Lagenaria siceraria*), etc. Compared to the total number of species in the Cucurbitaceae family, the number of purified lectins is only a few [11,13,14].

The isolation of a lectin from *Cucurbita pepo* was first reported in 1979 by Allen [15]. Though its activity was weakly inhibited by *N*-acetylglucosamine and its methyl glycosides, lectin has a much stronger specificity for chitin oligosaccharides. Later, several PP2-type CPELs and proteins were purified by different chromatographic methods, and their amino acid sequences have also been compared. It was observed that 100-Leu-Ile-Glu-Val-Ser-Trp-105 and 199-Trp-Lys-200 amino acid residues were highly conserved among these proteins and responsible for binding to chitooligosaccharides. The functional roles of CPELs in plants included chitin-binding properties, anti-pathogenic response, wound-sealing, RNA-binding and transport as well as molecular chaperone-like activity in the stress response [11,16]. 

In 1979, the lectin was isolated using a triacetyl chitotriose–sepharose column consisting of a single polypeptide chain of 20 kDa [15]. Ravichandran et al. reported the purification of another squash lectin through ammonium sulfate precipitation, but they did not determine its molecular weight and only assumed it to be more than 12 kDa [13]. In this study, an acetylated chitin column was used to purify the *Cucurbita pepo* lectin (denoted as CPL) with an estimated molecular weight of 30 ± 1 kDa. Swamy et al. and others reported CPELs (Cucurbita phloem exudate lectins) to be homodimeric proteins with subunits of ~16–26 kDa molecular mass proteins [11,16,17,18,19,20]. Phloem exudate proteins with various molecular weights were purified long ago from *Cucumis melo*, *Cucumis sativus* and *Cucurbita maxima* [21]. The partial purification of a lectin from *Praecitrullus fistulosus* (Round gourd) has also been reported [22]. All these lectins were chitooligosaccharide ([(GlcNAc)3-6])-specific, which, for us, was the reason behind using a chitin column to purify the lectin. However, it was revealed that CPL possessed greater affinity to 4-nitrophenyl-α-D-glucopyranoside, lactose and rhamnose than *N*-acetyl-D-glucosamine, the monomer of chitin. However, it cannot be compared as the sugar specificity of other CPELs was not studied in detail.

Despite all this progress in the last five decades, there is no report on the biological activities of *Cucurbita pepo* phloem exudate lectins. As the anti-parasitic and antifungal activities of lectins from *Cucurbita pepo* phloem exudate have already been predicted due to their chitin-binding properties, we report their effects on pathogenic microorganisms, fungi and cancer cells for the first time.

## 2. Results

### 2.1. Purification of the Lectin and Its Minimum Hemagglutination Activity for Mice Erythrocytes

**Figure 1:**
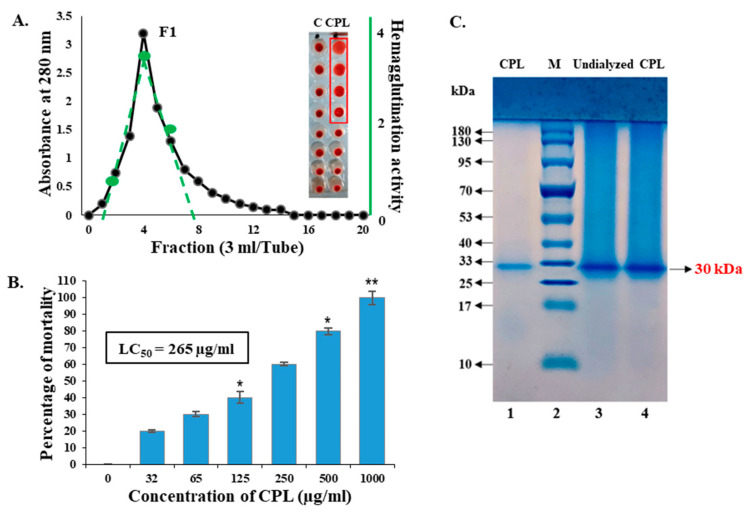
Purification, hemagglutination activity and toxicity of CPL. (**A**) Purification of CPL by affinity chromatography. The minimum concentration of CPL required to agglutinate mice erythrocytes was 31.25 μg/mL. The green line indicates the lectin fraction with hemagglutinating activity. The red frame shows the hemagglutination titer of CPL. (**B**) SDS-PAGE of purified CPL. Lane 1: purified CPL. Lane 2 (M): standard markers of phosphorylase b (94 kDa), serum albumin (66 kDa), ovalbumin (42 kDa), carbonic anhydrase (30 kDa), trypsin inhibitor (21 kDa) and lysozyme (14 kDa). Lane 3 and 4: undialyzed CPL. (**C**) Toxicity of CPL against brine shrimp nauplii. Results are presented as means ± SD (*n* = 3). Statistical significance was determined with *p* < 0.01, with the data showing significance denoted as * *p* < 0.05 and ** *p* < 0.01.

A chitin-binding lectin from squash fruits (designated as *Cucurbita pepo* Lectin or CPL) was isolated using affinity chromatography. An F1 fraction with significant hemagglutinating activity was applied in SDS-PAGE and a single band of protein (CPL) was obtained. Four wells of serially diluted solutions of CPL in the microtiter plate showed agglutinating activity with mice erythrocytes (Figure 1A). The minimum hemagglutination concentration was determined to be 31.25 µg/mL. The estimated molecular weight of the unknown chitin-binding lectin (CPL) was 30 ± 1 KDa (Figure 1B). The purification profile of CPL is shown in Table 1. This single-step purification process was less time-consuming. Though the yield decreased significantly, the lectin was satisfactorily purified with a purification factor of 9.09-fold.

### 2.2. Toxicity of CPL against Brine Shrimp Nauplii

The toxic effect of CPL against brine shrimp nauplii was determined after 24 h of treatment. The mortality rate of brine shrimp nauplii rose with increasing concentrations of CPL, and 2–10 nauplii died at the concentration range of 32–1000 µg/mL. The estimated LC_50_ value was 265 µg/mL as determined by probit analysis (Figure 1C).

### 2.3. Effect of Temperature and pH on the Hemagglutination of CPL

**Figure 2:**
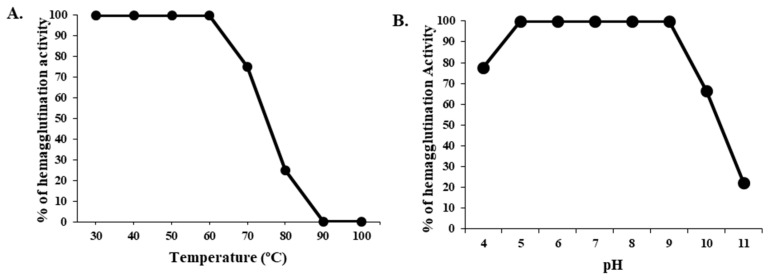
Effect of temperature and pH on CPL hemagglutination activity. (**A**) CPL thermostability. (**B**) Influence of pH on CPL hemagglutination activity. For both experiments, the results are presented as the mean ± standard deviation (*n* = 3), with error bars indicating the standard error from the triplicate trials.

At a range of temperatures from 30 °C to 100 °C, the hemagglutination activity of CPL was tested for 30 min. The lectin was 100% active until the temperature reached 60 °C. After that point, its hemagglutination activity started to decrease, and above 80 °C, its activity decreased to 75%. CPL became completely inactive at 90 °C (Figure 2). At the pH range of 5.0–9.0, CPL was fully active, but at pH 4.0 and 10.0, the lectin lost 22.23% and 33.34% of its activity. The protein lost 80–100% of its activity at pH 11.0 or higher. 

### 2.4. Effect of Denaturants and Divalent Metal Ions on the Activity of CPL

After the treatment of CPL with 1 M, 2 M and 3 M urea for 2 and 4 h, its hemagglutination activity did not decrease compared to the control. CPL lost 20% and 30% of its hemagglutination activity after treatment with 50 and 100 mM of EDTA. It recovered full hemagglutination activity when treated with 20 mM of Ca^2+^, which did not happen when treated with Mg^2+^, Mn^2+^ and Zn^2+^ (Table 2).

### 2.5. Hemagglutination Inhibition Study of CPL by Various Sugars

The hemagglutination activity of CPL became strongly inhibited in the presence of 4-nitrophenyl-α-D-glucopyranoside, lactose, rhamnose and *N*-acetyl-D-glucosamine (the monomer of chitin). Raffinose, galactose and fucose also somewhat inhibited the activity (Table 3).

### 2.6. Bactericidal and Antibiofilm Activity of CPL

**Figure 3:**
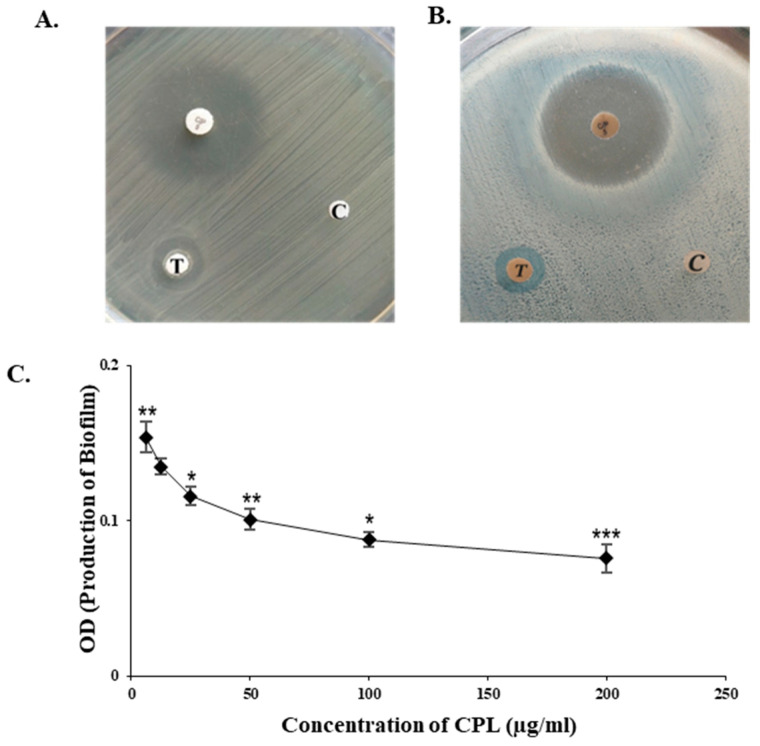
Bactericidal and antibiofilm properties of CPL. Antibacterial effectiveness of CPL against (**A**) *Escherichia coli* (ATCC 27853) and (**B**) *Shigella dysenteriae* (ATCC 238135). In this context, T and C indicate the sample disc with a high concentration of CPL (100 µg/disc) and the control disc (without CPL), respectively. A standard antibiotic disc (ciprofloxacin 5 µg) is used in both tests. (**C**) Antibiofilm activity of CPL against *E. coli*. Error bars: data represent the mean ± SD (*n* = 3) from triplicate experiments, with standard errors (SEs) shown. Statistical significance is noted as *p* < 0.01, with data represented as * *p* < 0.05, ** *p* < 0.01 and *** *p* < 0.001 to indicate the levels of significance.

CPL exhibited noticeable bactericidal activity against all the bacteria tested, though only the results against gram-negative bacteria *Escherichia coli* (ATCC 27853) and *Shigella dysenteriae* (ATCC 238135) compared to standard antibiotic (ciprofloxacin) and control discs are shown here (Figure 3A,B). CPL inhibited 57.06% of the formation of biofilm by *Escherichia coli* at 200 µg/mL (Figure 3C). 

### 2.7. Bacteriostatic Activity of CPL against Different Pathogenic Bacteria

**Figure 4:**
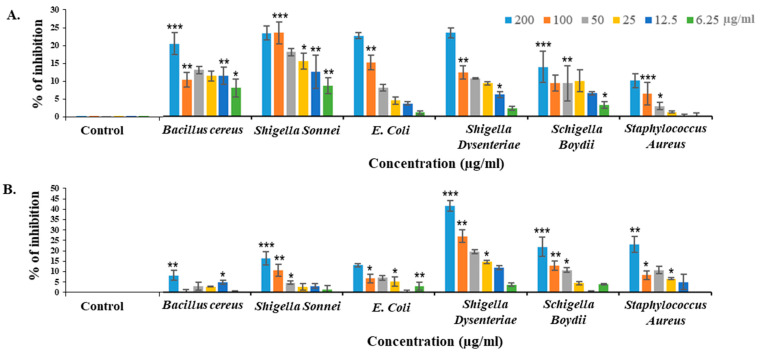
(**A**) Bacteriostatic effects of CPL on *Bacillus cereus* (ATCC 14579), *Shigella sonnei* (ATCC 29930), *E. coli* (ATCC 27853), *Shigella dysenteriae* (ATCC 238135), *Shigella boydii* (ATCC 231903) and *Staphylococcus aureus* (ATCC 25923) at concentrations ranging from 6.25 to 200 µg/mL. (**A**) Results after 24 h of treatment. (**B**) Results after 48 h of treatment. The graph uses blue, orange, black, yellow, dark blue and green to represent different CPL concentrations (200, 100, 50, 25, 12.5 and 6.25 µg/mL, respectively). For each bacterium, there were one negative control and six positive controls. Results are presented as means ± SD (*n* = 3). Statistical significance was determined at *p* < 0.01, with data indicated as * *p* < 0.05, ** *p* < 0.01 and *** *p* < 0.001.

CPL inhibited the growth of pathogenic bacteria in a dose-dependent manner. It showed 24.29% and 25.11% growth suppressive effects against *Escherichia coli* (ATCC 27853) and *Shigella dysenteriae* (ATCC 238135) after 24 h. However, the activity dropped to 10.76% against *Shigella boydii* (ATCC 231903) at a concentration of 200 µg/mL. After 48 h of treatment, CPL dramatically lost the bacteriostatic activity except *Shigella dysenteriae,* which showed 44.53% growth inhibition, even after 48 h of treatment (Figure 4).

### 2.8. Antifungal Activity of CPL

**Figure 5:**
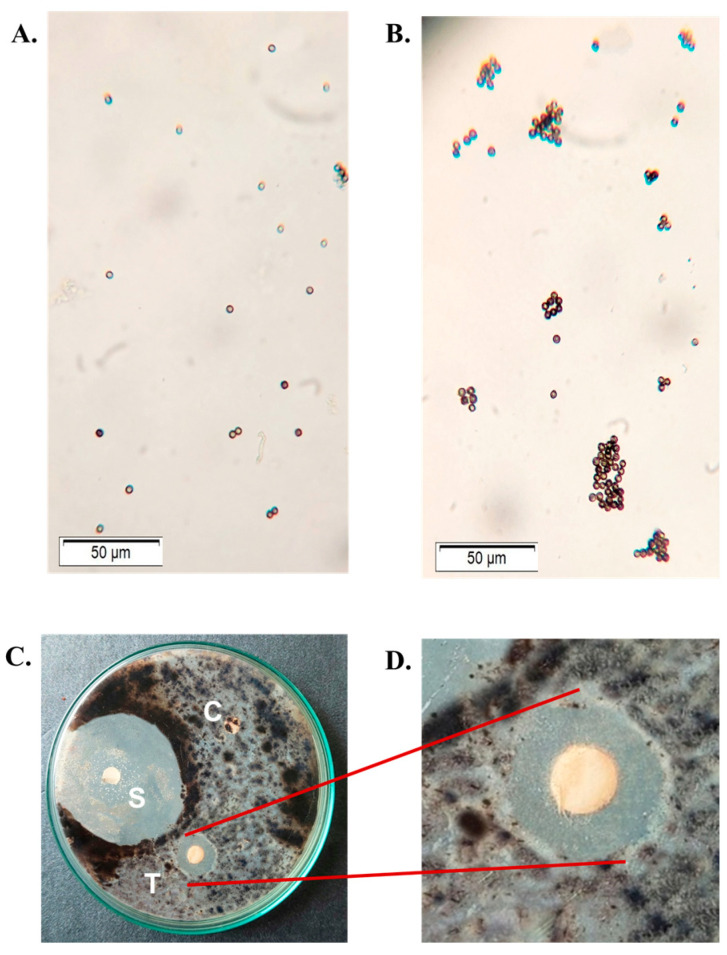
Antifungal activity of CPL. (**A**) Lectin-untreated spores of *Aspergillus flavus.* (**B**) Spores of *Aspergillus flavus* agglutinated by 100 µg/mL of CPL. (**C**) Disc diffusion assay demonstrating the fungistatic effect of CPL on *Aspergillus niger*. C: negative control disc (soaked with TBS only), T: test disc (200 µg/disc of CPL), S: positive control disc with 1% clotrimazole. (**D**) Magnified view of the test disc (T). Scale bar: 50 µm.

CPL agglutinated the *Aspergillus flavus* spores at a concentration of 100 µg/mL (Figure 5B), whereas no agglutination was observed for untreated spores (Figure 5A). It suppressed the growth of pathogenic fungus, *Aspergillus niger*, at a concentration of 200 µg/disc. The activity was determined by measuring the zones of inhibition formed by the control disc, the CPL-treated disc and the disc with a standard antifungal agent (1% clotrimazole) (Figure 5C). 

### 2.9. Agglutination of Ehrlich Ascites Carcinoma (EAC) Cells by CPL and Its In Vivo Anticancer Activity

**Figure 6:**
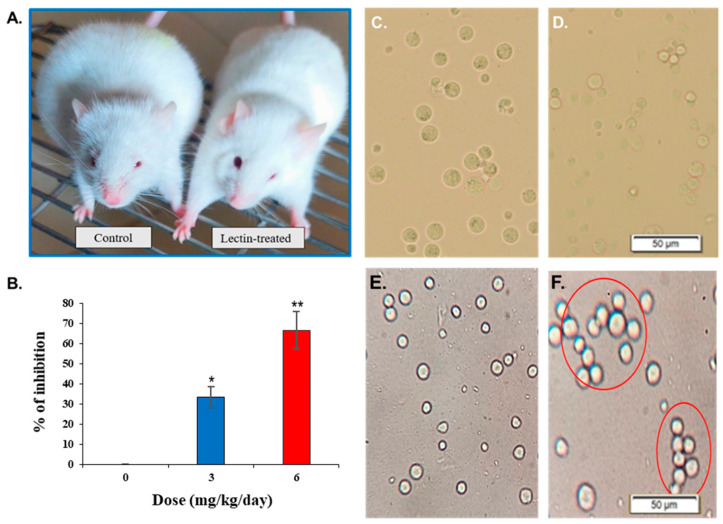
Anticancer activity (in vivo) of CPL against EAC cells. (**A**) Lectin-untreated (control) and CPL-treated Swiss albino mice. (**B**) In vivo inhibitory effects of CPL on EAC cell growth at low (3 mg/kg/day) and high (6 mg/kg/day) doses compared to control mice. Data are presented as means ± SD (*n* = 3). Number and appearance of (**C**) lectin-untreated and (**D**) CPL-treated EAC cells. (**E**) EAC cells from control mice. (**F**) Agglutination of lectin-treated EAC cells. Scale bar: 50 µm. Here, *p* < 0.01 was considered to be statistically significant and the data are represented as * *p* < 0.05 and ** *p* < 0.01 to indicate the levels of significance.

With increasing doses of CPL, the proliferation of Ehrlich Ascites Carcinoma (EAC) cells became severely suppressed. The growth inhibition of EAC cells in vivo by CPL was 36.44% and 66.66% at doses of 3 mg/kg/day and 6 mg/kg/day, respectively (Figure 6B). Compared to the untreated or control mice (Figure 6C), the number of EAC cells significantly decreased in squash lectin-treated mice (Figure 6D). CPL agglutinated EAC cells at a concentration of 50 µg/mL (Figure 6F), but this activity was absent in the control or untreated EAC cells (Figure 6E). 

### 2.10. Examination of the Morphology of Lectin-Treated and Untreated EAC Cells

**Figure 7:**
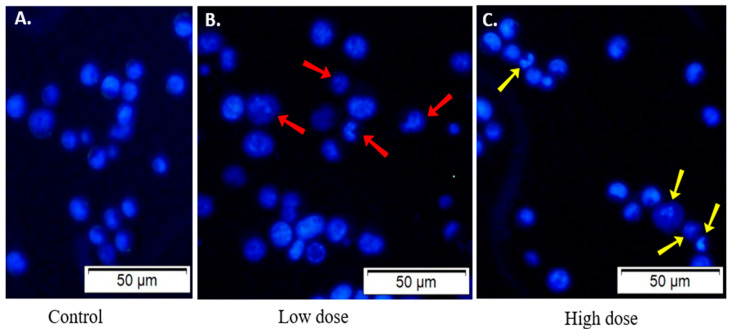
Morphology of CPL-treated EAC cells. (**A**) EAC cells from control mice exhibit a regular round shape. Treated EAC cells display irregular size and shape, with apoptotic morphological changes evident at (**B**) low dose (3 mg/kg/day) and (**C**) high dose (6 mg/kg/day) of CPL. Scale bar: 50 µm.

Both CPL-treated and untreated (control) EAC cells were subjected to Hoechst-33342 staining to monitor the morphological alterations. The nuclei of control EAC cells were regular-sized, round-shaped and homogeneously stained when viewed in a fluorescence microscope (Figure 7A). In contrast, CPL-treated cells were irregular in size and shape. These cells showed characteristic morphological changes (e.g., membrane blebbing, cell shrinkage, chromatin condensation and nuclear fragmentation) when observed in a fluorescence microscope at low (Figure 7B) and high (Figure 7C) doses.

### 2.11. Effect of CPL on Blood Parameters of EAC-Bearing and Normal Mice

**Figure 8:**
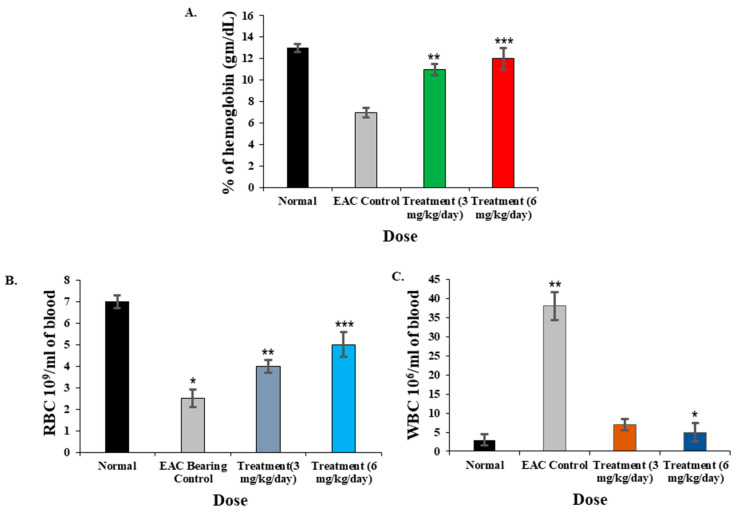
Impact of CPL on blood parameters in EAC-bearing (control) and normal mice. (**A**) Hemoglobin percentage in CPL-treated mice compared to EAC-bearing control and normal mice. (**B**) RBC count increased in CPL-treated mice with rising lectin concentrations (3 to 6 mg/kg/day) relative to control and normal mice. (**C**) WBC count significantly decreased in lectin-treated mice compared to control mice, approaching levels observed in normal mice. Results are shown as means ± SD (*n* = 3). Here, *p* < 0.01 was considered to be statistically significant and the data are represented as * *p* < 0.05, ** *p* < 0.01 and *** *p* < 0.001.

After the treatment of EAC mice with lectin for twelve consecutive days, blood parameters of the treated mice significantly improved. The hemoglobin percentage of the mice treated with 3 and 6 mg/kg/day of CPL increased significantly compared to the control mice receiving no treatment (Figure 8A,B) and almost reached the normal level. The total RBC count also increased twice in lectin-treated mice comparing to the control and got close to the value of normal mice. Compared to the control, the WBC count significantly decreased in lectin-treated mice and was almost restored to the normal level (Figure 8C).

### 2.12. In Vitro Antiproliferative Activity of CPL against Human Cancer Cell Lines (MCF-7 and A-549) by MTT Assay

**Figure 9:**
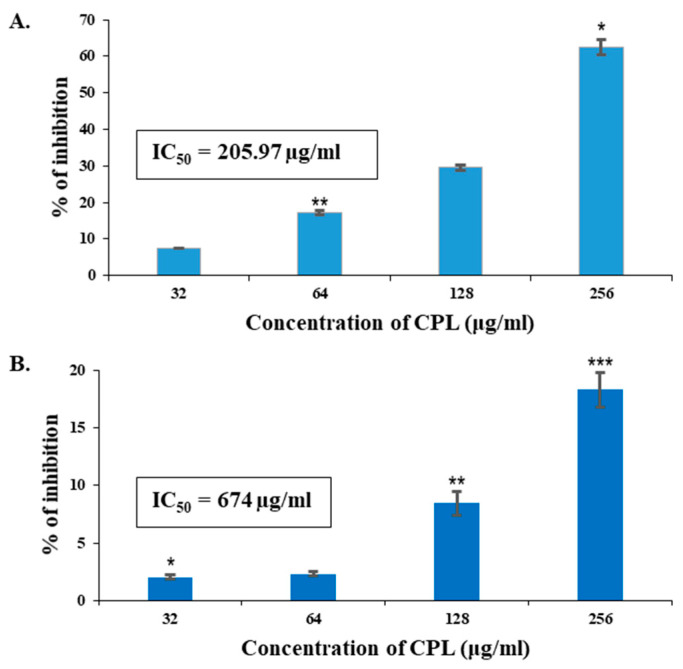
In vitro antiproliferative activity of CPL against human cancer cell lines. (**A**) CPL exhibited significant antiproliferative effects on human breast cancer cells (MCF-7) within the concentration range of 32–256 µg/mL. (**B**) CPL demonstrated milder antiproliferative effects on the human lung cancer cell line (A-549). Results are presented as means ± SD (*n* = 3). Here, *p* < 0.01 was considered to be statistically significant and the data are represented as * *p* < 0.05, ** *p* < 0.01 and *** *p* < 0.001.

Significant (7.58–62.54%) growth inhibitory activity against MCF-7 cells was exhibited by CPL in a dose-dependent manner (Figure 9A), though lower values (2.07–18.3%) were found against A-549 (human lung cancer cell line) (Figure 9B). The IC_50_ values of CPL against MCF-7 and A-549 were 205.97 µg/mL and 674.0 µg/mL, respectively.

## 3. Discussion

In this discussion, we focused on lectins from the Cucurbitaceae family due to the insufficiency of relevant data on CPELs. CPL was stable at 30 to 60 °C and 5.0 to 9.0, whereas the activity of *Momordica charantia* (Bitter gourd) seed lectin (MCL) was maximal at 4–50 °C but decreased abruptly below pH 7.0 [23]. Similar to CPL, the lectin from *Trichosanthes dioica* (Pointed gourd) seeds (TDSL) was also active until the temperature and pH reached above 60 °C and 10.0, respectively [24]. But, *Trichosanthes cucumerina* (Snake gourd) seed lectin (TCSL) and *Praecitrullus fistulosus* (Round gourd) lectin (PfLP) demonstrated high agglutination activity at the temperature range 30–90 °C and 0–80 °C. TCSL and PfLP did not lose their activity at pH 3.0–12.0 and 2.0–9.0 [25]. Plant lectins typically exhibit resilience to elevated temperatures when heated [8,26]. However, few of them are stable at high temperatures [27]. 

Research has shown that lectins rely on metal ions for their functions, and certain metal ions assist in preserving the lectin structure and stabilizing amino acid residues at particular sugar side-chain binding sites. CPL lost 20% and 30% of hemagglutination activity when treated with 50 mM and 100 mM EDTA, respectively, but recovered it in the presence of metal cations like Ca^2+^, Mg^2+^, Al^3+^ and Fe^3+^. In contrast, TCSL was metal-independent for its activity [25]. Tough CPL remained stable when treated with denaturants and retained its activity in the presence of 3M urea, and it experienced a partial loss of activity when treated with EDTA. 

Phloem-based defense is a common plant mechanism against pathogen invasion. Phloem proteins 1 and 2 (PP1 and PP2) crosslink to seal wounds and block the entry of pests and insects. PP2 interacts with N-linked glycans, chitin and chitooligosaccharides—major components of insect exoskeletons and fungal cell walls [28,29]. Reasonably, PP2 lectins can deter pathogens in plants. Therefore, the toxicity and antimicrobial and anticancer activities of CPL were sequentially examined.

The brine shrimp lethality assay showed CPL’s impact on the mortality rate of brine shrimp. At the concentration of 125 µg/mL of CPL, the mortality rate was 40% but increased to 100% when the concentration was raised to 1 mg/mL, with an LC_50_ value of 265 µg/mL. The LC_50_ values of TCSL (*Trichosanthes cucumerina* seed lectin), TDSL (*Trichosanthes dioica* seed lectin) and MCL (*Momordica charantia* lectin) against brine shrimp nauplii were 261, 84 and 49.7 µg/mL, respectively [24,25,30]. 

Microorganisms often display surface carbohydrates that plant lectins can target [31]. For instance, the lipopolysaccharide of *Shigella* contains O-antigen backbones consisting of a repetition of three rhamnoses and one *N*-acetylglucosamine. *E. coli*’s peptidoglycan layer features *N*-acetyelglucosamine and *N*-acetyl muramic acid [32]. Given CPL’s ability to bind rhamnose and *N*-acetyelglucosamine (according to Section 2.5), it possibly exerted bactericidal activity against those Gram-negative bacteria, though the exact mechanism remains unclear. Another gourd lectin demonstrated agglutination capabilities against both Gram-positive (*Staphylococcus aureus*) and Gram-negative bacteria (*Salmonella enteritidis* and *Shigella flexneri*) at the minimum concentration of 50 µg/mL, also partially inhibiting the growth of *Salmonella enteritidis* and *Staphylococcus aureus* [33]. CPL showed varying degrees of bacterial growth inhibition against different pathogenic bacteria, with *Shigella dysenteriae* being notably sensitive to CPL even after 48 h. A study on the typing of *Shigella dysenteriae* strains by different lectins also indicated that *Shigella dysenteriae* strains are particularly susceptible to lectins like CPL, which specifically interacts with *N*-acetylglucosamine, galactose and fucose sugars [34]. Additionally, the lectin significantly (57.06%) suppressed biofilm formation by *E. coli* at 200 µg/mL. Recently, the antibiofilm activity of an aqueous extract from *Cucurbita pepo* was determined against *Streptococcus pyogenes*, but no other lectin from this plant family was reported to possess such activity [35]. 

Several proteins including PR-1, PR-2, PR-5, Cucurmoschin, Hispin, α-Momorcharin, Luffacylin and vicilin-like proteins from plants of the Cucurbitaceae family showed antifungal activity. They are classified as pathogen-related (PR) proteins, ribosomal inactivating proteins (RIPs), vicilin-like proteins and others [36]. But, no antifungal lectin from the phloem exudate of plants from this family has been reported so far. CPL exhibited strong antifungal activity against *Aspergillus niger* at a concentration of 120 µg/mL and agglutinated *Aspergillus flavus* spores at a concentration of 180 µg/mL. TCSL, a seed lectin was challenged against *Candida albicans*, *Aspergillus niger*, *Fusarium vasinfectum* and *Mucor* sp., but no antifungal activity of this lectin was detected [25]. 

Cancer is the leading cause of death worldwide after cardiovascular diseases. Studies reported that many plant-derived lectins are being used in cancer diagnosis and therapy [9]. Numerous plant lectins have been thoroughly examined for their in vivo anticancer potential against Ehrlich ascites carcinoma (EAC) cells in Swiss albino mice. In this study, CPL caused 36.44% and 66.66% of the growth inhibition of Ehrlich ascites carcinoma (EAC) when intraperitoneally administered at doses of 3 and 6 mg/kg/day, respectively. Compared to CPL, TCSL (28% and 72% at doses of 1 and 2 mg/kg/day), TDSL (7%, 50.2% and 60.3% at doses of 0.75, 1.5 and 3 mg/kg/day) and MCL (28%, 45% and 75% at doses of 1.2, 2.0 and 2.8 mg/kg/day) exerted stronger in vivo antiproliferative activity against EAC cells, though TDSL and MCL are more toxic than CPL [24,25,30]. Both TDSL and CPL agglutinated EAC cells, but morphological changes marked in CPL-treated EAC cells were absent when treated with MCL [24,30]. It also became evident that the intraperitoneal administration of three doses of PfLP (10 mg/kg) after tumor transplantation showed an effective antitumor response, reducing the EAC cell proliferation by 75% [22]. 

WBC count generally rises in tumor-bearing mice, but anemia is caused by a decrease in RBC or hemoglobin percentage due to hemolytic or myelopathic disorders, iron shortage, or both [37,38]. CPL showed protective effects in EAC-bearing mice on hematological parameters. The lectin increased the hemoglobin level and RBC count in treated mice and brought them back to the levels of normal mice. It also significantly decreased the WBC count when compared to the tumor-bearing mice. This result was very much in line with the previous results obtained by TCSL, TDSL and MCL, all three seed lectins from the Cucurbitaceae family [24,25,30].

The O-GlcNAcylation level increases in breast and colorectal cancer tissues [39,40], and a decrease in O-GlcNAcylation affects the malignant transformation of MCF-7 cells [41]. From another study, it became evident that the presence of L-fucose sugar could induce the binding ability of lectins [42]. In this study, CPL, a GlcNAc and fucose-binding lectin, induced a significant growth inhibition of MCF-7 cells in a dose-dependent manner. With an IC_50_ value of 205 µg/mL, it inhibited 29.53% of cancerous growth at the concentration of 128 µg/mL. In contrast, only a 7% growth inhibitory effect of CPL was observed against the lung cancer (A-549) cell line. Though the level of O-GlcNAcylation increases in A-549 cells as well, it happens only in response to glucose deprivation [43]. Perhaps this is the reason why CPL was comparatively more effective against MCF-7 cells than A-549 cells. A partially purified lectin (PfLP) from the phloem exudate of *Praecitrullus fistulosus* (Round gourd) exhibited antitumor activity against MCF-7 cells with an IC_50_ value of 62.80 µg/mL [22]. 

## 4. Materials and Methods

### 4.1. Materials

DMEM media, fetal bovine serum and Hoechst-33342 dye were bought from Sigma-Aldrich (St. Louis, MO, USA). A triple antibiotic mixture containing neomycin, penicillin and streptomycin were bought from CarlRoth GmbH (Karlsruhe, Germany). A standard protein marker mix was bought from Takara Bio Inc., Japan. (Kyoto, Japan). All other chemical/reagents including chitin powder were of the highest purity grades and bought from Wako Pure Chemical Co. (Osaka, Japan) and Sigma-Aldrich (St. Louis, MO, USA).

### 4.2. Purification of Cucurbita Pepo Lectin (CPL)

During the winter season (October–February), squash fruits were collected from a local market in Rajshahi City, Bangladesh. The fruits were cleaned, crushed and blended to prepare the crude sample, which was then stored at 0 °C. The acetylation of chitin was conducted by following the procedure from Nishi et al., 1979, with few modifications [44,45]. The final product, acetylated chitin, was filtered, washed with ethanol, filtered again and packed in a column washed with 20 mM Tris-HCl buffer (pH 8.2). The crude protein was subjected to this column which was then washed with the same buffer. After eluting the chitin-binding lectin with 0.5 M acetic acid, the protein fraction was immediately dialyzed overnight with 20 mM Tris-HCl buffer (pH 8.2) and ice-cold deionized water. After dialysis, the protein was concentrated by aquacide II powder and stored at 0 °C for further use.

### 4.3. Hemagglutination Activity of CPL

One ml of blood from Swiss albino mice was collected in 1% NaCl solution and centrifuged at 2000 rpm for 5 min to isolate red blood cells (RBCs). This process was repeated twice and each time the supernatant was discarded. RBCs were diluted with 1% NaCl solution to obtain the 2% RBC solution. Fifty µL of hemagglutination buffer (20 mM Tris-HCl buffer, containing 1% NaCl and 10 mM CaCl_2_, pH 7.8) was added to each well of the microtiter plate. After adding the lectin (CPL) to the first well, serial dilution was performed. Finally, 50 µL of 2% RBC solution was poured into each well. A titer plate shaker was used to shake the plate which was then incubated at 34 °C for 60 min to check the hemagglutination activity.

### 4.4. Brine Shrimp Lethality Bioassay

To prepare artificial seawater, 38 g of NaCl was dissolved in 1 L of distilled water and its pH was adjusted to 7.0 by adding sodium tetraborate salt. One gram of *Artemia* cysts were incubated in 1 L of seawater in a container at 28 ± 1 °C under constant light and aeration from the bottom of the container. After incubating for 48 h under these conditions, the cysts hatched. Various concentrations (32–1000 μg/mL) of CPL were taken in vials containing the seawater, and to each vial, 10 *Artemia* nauplii were added. The volume of each vial was adjusted to 4 mL by adding seawater. The experiments were conducted in triplicates. A test tube with 10 nauplii and no added lectin was taken as the negative control. The number of dead nauplii in each vial was counted after 24 and 48 h. Probit analysis software was used to determine the corresponding LC_50_ values [46].

### 4.5. Effect of Temperature and pH on Hemagglutination Activity of CPL

Moreover, 1 mg/mL of CPL was heated for 30 min at various temperatures (10–100 °C) in a water bath and cooled at room temperature to determine its thermal stability. Fifty µL of hemagglutination buffer (pH 7.8) was serially diluted with the same volume of CPL. The hemagglutination titer was determined by comparing it to a non-heated lectin sample (control) which denoted 100% activity. Furthermore, 1 mg/mL of the lectin solution was incubated against different buffers with pH values ranging from 4 to 11 for 6 h at room temperature. The dialysis of the lectin solutions was performed against 20 mM tris–HCl buffer (pH 8.2) for 12 h. Buffers used for the pH stability assay were as follows: 0.1 M sodium acetate (pH 4–6), 0.1 M phosphate (pH 7), 0.1 M Tris-HCl (pH 8.2) and 0.1 M glycine-NaOH (pH 9–11).

### 4.6. Treatment of CPL with Urea and Guanidine HCl

CPL (1 mg/mL) was incubated with urea (1 M, 2 M and 3 M) for 2 and 4 h to perceive the effect of the denaturants. CPL with 100% activity (containing no denaturant) was used as a control. After adding CPL to the first well of titer plate, it was serially diluted and 50 µL of RBC in saline (2%) was poured into all the wells. The plate was shaken for 5 min and incubated at 30 °C for 60 min [47].

### 4.7. Treatment of CPL with EDTA and Divalent Cations

One mg/mL of CPL was incubated in the presence of EDTA (50 and 100 mM) for 4 h at 30 °C. After dialyzing overnight with the Tris–HCl buffer (pH 8.2), the hemagglutination activity of CPL lectin was performed in the presence and absence of 20 mM Ca^2+^, Mn^2+^, Mg^2+^ and Zn^2+^ solutions to know its dependence on divalent metal cations. CPL with no EDTA was taken as a control. This activity was compared to that of control. Three replicates were used for each experiment.

### 4.8. Hemagglutination Inhibition Study of CPL by Various Sugars

Several simple and complex sugars (rhamnose, fucose, raffinose, *N*-acetyl-D-glucosamine, *N*-acetyl-D-galactosamine, Methyl-β-D-galactopyranoside, Methyl-α-D-mannopyranoside, glucose, galactose, mannose, D-Melibiose, lactose, D(+) xylose, 4-Nitrophenyl-β-D-galactopyranoside, 2-Nitrophenyl-β-D-galactopyranoside, 4-Nitrophenyl-α-D-glucopyranoside and 2-Nitrophenyl-α-D-mannopyranoside) were used to accomplish the hemagglutination inhibition test. Twenty five µL of hemagglutination buffer was added to every well of the microtiter plate. The same volume of the sugar solutions was then taken into the first well of the microtiter plate. After serial dilutions, CPL (25 µL, 1 mg/mL) and 50 µL of 2% RBC (in saline water) were added to all the wells. The plate was shaken as mentioned above [47].

### 4.9. Antimicrobial Activity of CPL

#### 4.9.1. Determination of Antibacterial Activity by Disc Diffusion Assay

Four bacterial species, *Bacillus cereus* (ATCC 14579), *Shigella boydii* (ATCC 231903), *Shigella sonnei* (ATCC 29930) and *Shigella dysenteriae* (ATCC 238135), were grown in nutrient broth and incubated at 37 °C overnight. The concentration of the bacterial solution was fixed by adjusting the optical density of the solution to 1.0 at 640 nm. Bacteria were spread out on sterilized petri dishes containing solidified nutrient agar media. A disc containing CPL (100 µg/disc), a standard antibiotic disc (5 µg/disc of ciprofloxacin) and a negative control disc (soaked with PBS) were prepared and placed on the Petri dishes. Bacterial cells were allowed to grow at 37 °C overnight. After 24 h, zones of inhibition were formed around CPL-containing discs. Those zones were measured and compared to the zones formed around the control and antibiotic discs.

#### 4.9.2. Determination of Antibiofilm Activity

The antibiofilm activity of CPL was studied according to our previous work [48]. In brief, *Pseudomonas aeruginosa* (ATCC 47085) was grown for 24 h, and the turbidity of bacterial cell suspensions was adjusted to 1.0 at OD_640_. The bacterial suspension (50 μL) was mixed with the same volume of CPL in 96-well microtiter plates and allowed to form the biofilm through incubation for 24 h at 37 °C. Crystal violet dye (0.1%) was used for 10 min to stain the biofilm formed in wells. The wells were washed with TBS to take out the free dye and 150 μL of 95% ethanol was added. After waiting 10 min, the absorbance values of each well were measured at 640 nm by a microtiter plate reader. The reduction in biofilm formation by CPL was calculated as follows:

% Reduction in biofilm formation = (1 − [OD_640_ experiment/OD_640_ control]) × 100%

#### 4.9.3. Determination of Bacteriostatic Activity against Different Pathogenic Bacteria

*Bacillus cereus* (ATCC 14579), *Shigella sonnei* (ATCC 29930), *E. coli* (ATCC 27853), *Shigella dysenteriae* (ATCC 238135), *Shigella boydii* (ATCC 231903) and *Staphylococcus aureus* (ATCC 25923) were grown in a liquid nutrient medium at 37 °C for 24 h and the concentration of bacterial solution was fixed by adjusting its optical density to 1.0 at 640 nm. CPL at the concentration range 6.25 to 200 µg/mL was added to a microtiter plate through serial dilutions, and 100 µL of bacterial suspension was added to a total volume of 200 µL in each well. Four wells were kept as control wells containing only bacteria and nutrient media. The plate was kept in an incubator for 24 h and absorbance values were recorded by a microtiter plate reader to calculate the percentage of growth inhibition.

#### 4.9.4. Fungistatic Activity of CPL against *Aspergillus niger*

The mycelia of a fungal strain, *Aspergillus niger*, was dissolved in distilled water and spread out on a sterile Petri dish containing potato dextrose agar. CPLs at various concentrations (200 µg/disc) and a standard antifungal agent (1% clotrimazole) were soaked out by the discs. The Petri dishes were then incubated at 30 °C until the growth of mycelia. Antifungal activity was observed by the formation of transparent rings around the discs.

#### 4.9.5. Agglutination of Fungal Spores by CPL

Spores of *Aspergillus flavus* were dissolved in phosphate buffer saline (PBS) and taken into two Petri dishes. CPL was added to one Petri dish at a concentration of 100 µg/mL, whereas the same amount of PBS was added to another. After 10 min, both Petri dishes were checked under a microscope to find any agglutination activity of CPL to fungal spores. 

### 4.10. Anticancer Activity of CPL 

#### 4.10.1. Culture of Ehrlich Ascites Carcinoma Cells In Vivo in Swiss Albino Mice and Study of the Anticancer Activity of CPL

Swiss albino male mice of 4–5 weeks of age, weighing 20–27 g, were collected from the Department of Pharmacy, Jahangirnagar University, Savar, Bangladesh. Twelve mice were distributed into three groups (four mice in each group)—low (3 mg/Kg/day) dose, high (6 mg/Kg/day) dose and control. Moreover, 5 mL of 1% saline water was injected intraperitoneally in 7-day-old ascites tumor-bearing mice. Before cell collection, mice were made unconscious by using chloroform, and EAC cells were collected from the peritoneal region of cancer-bearing mice. Then, the EAC cell suspension was diluted using an isotonic solution (1% saline) to achieve 1,000,000 cells per mL. The number of cells was adjusted by counting them under a microscope. Moreover, 1 mL of this cell suspension was taken in a 1 mL syringe, and 100 µL EAC cell suspension (3 × 10^6^ cells/mL) was injected into each of the mice. After 24 h, CPL was injected intraperitoneally in the groups for high and low doses for 5 days. On the 6th day, the mice were sacrificed, and intraperitoneal EAC cells were collected and harvested in normal saline. EAC cells were counted by a hemocytometer, and the total number of viable cells in every lectin-treated mice group was compared with those of the controls (untreated, containing only EAC cells). The percentage of growth inhibition was estimated using the following formula:

% of growth inhibition = 100 − {(Cells from CPL treated mice/Cells from control mice × 100}

#### 4.10.2. Determination of Hematological Parameters of Normal, EAC-Bearing and Lectin-Treated Mice

Eighteen mice were divided into three groups to perform the experiments. Six were kept as ‘Control’ (EAC-bearing but untreated) and ‘Normal’ (non-EAC-bearing). The rest of the mice were treated with high and low doses of CPL as mentioned above. On the 12th day of EAC inoculation, 50 µL of blood was collected from the hearts of unconscious mice and was poured in an Eppendorf tube containing 2 µL of EDTA. EDTA was used to prevent the clotting of blood. Then, 10 µL of EDTA-containing blood was taken in 1 mL RBC diluting fluid in one Eppendorf tube for each mouse. And, 100 µL blood suspension was diluted again with 900 µL RBC diluting fluid. Finally, 10 µL of suspension was taken and the cell number was counted under a microscope. 

Ten µL blood was previously mixed with 2 µL EDTA, and 1000 µL of WBC diluting fluid was mixed in another Eppendorf tube for each mouse. Then, the hemocytometer and cover slip were washed with ethanol and cleaned with tissue paper. After that, the cover slip was placed on the counting chamber, and the blood suspension was poured between the cover slip and chamber through a micropipette. For WBC count, the squares of the 4 corners of the hemocytometer were counted.

Two hundred µL of 0.1N HCl solution was taken in Hb diluting tubes. Then, 20 µL blood was added to the Hb diluting tube and the mixture was turned into a brown color. The diluting tube was placed in a hemometer, and distilled water was added to the tube drop by drop and gradually mixed. The color of the mixture was compared with the color of the prism. After matching the color, the concentration of Hb present in the blood was calculated.

#### 4.10.3. Observation of Morphologic Changes and Nuclear Damages of EAC Cells through Fluorescence Microscope

After the treatment of Swiss albino mice with CPL (at 3 and 6 mg/kg/day) and without CPL, EAC cells were collected from the mice using the same procedure described above and washed three times with PBS. Hoechst 33,342 dye (0.1 µg/mL) was used to stain these cells and kept for 20 min in the dark at 37 °C. To remove the free dye, PBS was used again to wash EAC cells. The apoptosis of EAC cells was morphologically observed under a fluorescent microscope.

#### 4.10.4. Determination of Antiproliferative Activity Assay (In Vitro) of CPL against Different Human Cancer Lines by MTT Assay

MCF-7 and A-549 cells were cultured in DMEM media with 10% FBS (penicillin, neomycin and streptomycin were used as antibiotics) using a 96-well flat-bottom culture plate at 37 °C in a 5% CO_2_ incubator. Subcultures were carried out at 80–90% confluence. In case of MCF-7, 1 × 10^4^ cells were seeded in 150 μL of DMEM media in each well of the plate and incubated at 37 °C in a CO_2_ incubator for 24 h. In case of A-459 cells, 2 × 10^4^ cells were seeded in each well containing 150 μL of DMEM media and incubated for 24 h. CPL was serially diluted in 50 μL of DMEM media and added to each well of the plate to final concentrations of 32–256 µg/mL and incubated again for 48 h.

Three wells were used as ‘control’ wells containing only EAC cells. The aliquot from each well was carefully discarded, then 180 µL of PBS and 20 µL of MTT were added to those. After incubating it again at 37 °C for 8 h, the aliquot was again discarded and acidic isopropanol (200 µL) was added into each well. After keeping it in the incubator at 37 °C for 1 h, absorbance values were recorded at 570 nm with a titer plate reader. The proliferation inhibition ratio of EAC cells was calculated as follows:

Proliferation inhibition ratio (%) = (A − B) ×100/A

where A is the OD at 570 nm of the cellular homogenate (control) without lectin and B is the OD at 570 nm of the cellular homogenate with lectin (CPL). 

### 4.11. Statistical Analysis

All experiments of this research work were performed in triplicates. The results were expressed as mean ± standard error (SEM). Here, *p* < 0.01 was considered to be statistically significant and the data are represented as * *p* < 0.05, ** *p* < 0.01 and *** *p* < 0.001. The statistical significance of data was evaluated by the one-way analysis of variance (ANOVA) and Student’s *t*-test with Graph Pad Prism 5.1. 

## 5. Conclusions

With their unique glycan recognition property, lectins are useful in the detection, isolation and characterization of glycoconjugates, tissue histochemistry and identification of tumor cells. This study highlights the multifunctional potential of *Cucurbita pepo* lectin (CPL). The lectin is stable across varied temperatures and pH levels and exhibited significant antimicrobial activities. As an antibiofilm agent, it can combat the multidrug-resistant pathogen-associated infections. CPL’s selective cytotoxicity against MCF-7 breast cancer cells and its positive effects on hematological parameters in EAC-bearing mice also underscore its therapeutic potential. Despite limitations such as the lack of N-terminal sequencing or mass spectrometry for molecular weight confirmation and detailed mechanistic studies, CPL’s broad bioactivity spectrum and mild toxicity mark it as a promising candidate for developing novel antimicrobial and anticancer therapies. Further investigation is necessary to fully explore its mechanisms of action and biomedical applications.

## Figures and Tables

**Table 1:** Purification profile of squash (*Cucurbita pepo*) lectin.

Purification Step	Total Protein (mg)	Total Activity (HU)	Specific Activity (HU/mg)	Purification Fold	% of Yield (Per Step)
Crude extract	1420	30,000	21.13	1	100
Affinity chromatography	38	7300	192.10	9.09	2.67

The given data are calculated for 100 g of squash fruits. Mice erythrocytes were used for the hemagglutination assay.

**Table 2:** Hemagglutination activity of CPL after demetallization and addition of metal ions.

Native Lectin	Demetallized Lectin	Demetallized Lectins after Reconstitution with Metal Ions			
		Ca^2+^	Mg^2+^	Mn^2+^	Zn^2+^
256 ± 0	0	256 ± 0	128 ± 0	128 ± 0	128 ± 0

Hemagglutination titer values are represented as mean ± SEM. At least three independent experiments were performed.

**Table 3:** Inhibition of the hemagglutination activity of CPL by various sugars (1–17). C denotes the negative control used in this experiment.

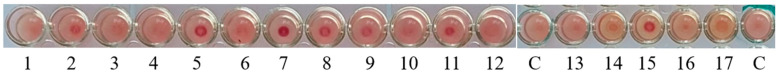							
Sl. no	Name of Sugar	Concentration (mM)	Degree of Inhibition	Sl. no	Name of Sugar	Concentration (mM)	Degree of Inhibition
1	Glucose	200	−	10	*N*-acetyl-D-galactosamine	200	−
2	Galactose	200	**+**	11	***N*** **-acetyl-D-glucosamine**	**200**	**++**
3	Mannose	200	−	12	Methyl-β-D-galactopyranoside	200	−
4	D-Melibiose	100	−	13	Methyl-α-D-galactopyranoside	200	−
5	**Lactose**	**100**	**+++**	14	4-Nitrophenyl-β-D-galactopyranoside	12.5	−
6	D (+) Xylose	200	−	15	**4-Nitrophenyl-α-D-glucopyranoside**	**6.25**	**++**
7	**Rhamnose**	**200**	**+++**	16	2-Nitrophenyl-β-D-galactopyranoside	12.5	−
8	Fucose	200	**+**	17	4-Nitrophenyl-α-D-mannopyranoside	5	−
9	Raffinose	100	**+**				

## Data Availability

Data are available in this article.
